# Nanomaterials-Incorporated Chemically Modified Gelatin Methacryloyl-Based Biomedical Composites: A Novel Approach for Bone Tissue Engineering

**DOI:** 10.3390/pharmaceutics14122645

**Published:** 2022-11-29

**Authors:** Abigail Herrera-Ruiz, Benjamín Betancourt Tovar, Rubén Gutiérrez García, María Fernanda Leal Tamez, Narsimha Mamidi

**Affiliations:** 1 Department of Chemistry and Nanotechnology, The School of Engineering and Science, Tecnologico de Monterrey, Monterrey 64849, Mexico; 2 Department of Chemical Engineering, The School of Engineering and Science, Tecnologico de Monterrey, Monterrey 64988, Mexico

**Keywords:** GelMA, nanoparticles, nanomaterials, carbon nanomaterials, bone tissue engineering

## Abstract

Gelatin methacryloyl (GelMA)-based composites are evolving three-dimensional (3D) networking hydrophilic protein composite scaffolds with high water content. These protein composites have been devoted to biomedical applications due to their unique abilities, such as flexibility, soft structure, versatility, stimuli-responsiveness, biocompatibility, biodegradability, and others. They resemble the native extracellular matrix (ECM) thanks to their remarkable cell-adhesion and matrix-metalloproteinase (MMP)-responsive amino acid motifs. These favorable properties promote cells to proliferate and inflate within GelMA-protein scaffolds. The performance of GelMA composites has been enriched using cell-amenable components, including peptides and proteins with a high affinity to harmonize cellular activities and tissue morphologies. Due to their inimitable merits, GelMA systems have been used in various fields such as drug delivery, biosensor, the food industry, biomedical, and other health sectors. The current knowledge and the role of GelMA scaffolds in bone tissue engineering are limited. The rational design and development of novel nanomaterials-incorporated GelMA-based composites with unique physicochemical and biological advantages would be used to regulate cellular functionality and bone regeneration. Substantial challenges remain. This review focuses on recent progress in mitigating those disputes. The study opens with a brief introduction to bone tissue engineering and GelMA-based composites, followed by their potential applications in bone tissue engineering. The future perspectives and current challenges of GelMA composites are demonstrated. This review would guide the researchers to design and fabricate more efficient multifunctional GelMA-based composites with improved characteristics for their practical applications in bone tissue engineering and biomedical segments.

## 1. Introduction

The natural process of bone regeneration may be affected by significant bone defects, patient co-morbidities, and inflammatory disorders, which decrease the self-healing capacity of the skeletal system [1]. In bone defects, the healing process can be divided into three main stages: (i) cartilage generation, (ii) biomineralization in the cartilage occurs, and (iii) bone formation. However, with severe bone defects, which physical accidents can cause, the regeneration process cannot occur without surgical interventions. A way to solve bone defects is with bone tissue engineering (BTE) [2]. Tissue engineering focuses on using biodegradable and porous scaffolds that allow cells to adhere and proliferate. Consequently, the necessary conditions are created for forming structures like the extracellular matrix (ECM) [3]. The process of BTE starts with the migration and recruitment of osteoprogenitor cells. Then, proliferation and differentiation of these cells occur, forming a matrix, and finally, bone remodeling occurs [4]. The natural bone tissue comprises macro, micro, and nanostructures made of organic and inorganic compounds [2]. The use of nanomaterials in scaffolds helps mimic the biological nanostructure of the ECM [4]. The existing different ways and strategies implemented in bone tissue engineering include biological, osteoinductive, and hybrid materials, as well as cell therapy [5]. Biocompatibility, mechanical qualities, and biodegradability are unique properties that make biomaterials one of the most suitable strategies for BTE. Several biomedical applications of biomaterials consist of joint replacements [6], contact lenses [7], drug delivery systems [8], tissue engineering [9], and others. Specifically, hydrogels can swell in water multiple times their original weight without dissolution. They resemble the natural ECM; hence they are optimal for recreating conditions for in vitro cell culture and can provide three-dimensional (3D) support for tissue formation [10,11]. Hydrogels are promising in repairing cartilage injuries, skull defects, and arthritis due to their tunable mechanical strength, favorable compatibility, and bioactivity. For this reason, they are considered good candidates for the BTE field [12].

Several hydrogels are developed for bone tissue engineering. Among them, methacryloyl functionalized gelatin (GelMA) is one of the famous biomaterials. GelMA is a derivative of gelatin. Likewise, gelatin is derived from collagen. This makes gelatin-based scaffolds great candidates for biomedical applications since collagen is the most abundant protein found in the skin’s bones, cartilage, and connective tissue [10]. Gelatin has multiple advantages over collagen, these include a lower antigenicity, higher solubility and it is less immunogenic. Gelatin is an attractive polymer since it is economically accessible and highly available. It preserves the bioactive groups of collagens that promote cell attachment and remodeling: the arginyl-glycine-aspartic acid (RGD) sequences and the matrix metalloproteinase (MMP) target sequences [13]. However, for the gelatin to be applied, it needs to be crosslinked since it is not stable at body temperature and in an aqueous system. Modification of gelatin with methacrylic groups is needed to synthesize GelMA. This allows crosslinking to form hydrogels with specific properties. The sequences of RGD and MMP are not affected by derivatization. Therefore, GelMA is a good choice for bone tissue engineering and regenerative medicine.

**Figure 1:**
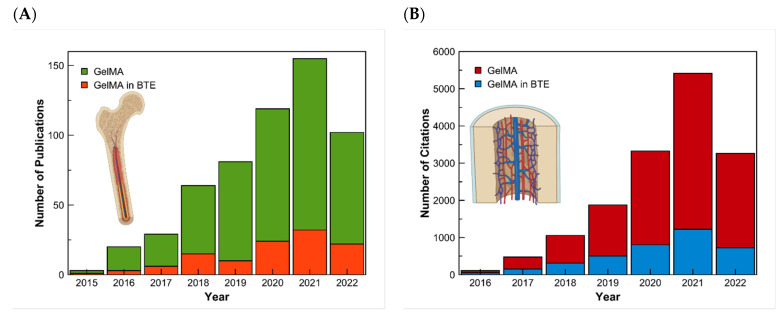
Research results in the Scopus Database for GelMA and GelMA in bone tissue engineering. (**A**) Results of the number of articles published, and (**B**) the number of citations from 2015 to 2022, respectively.

There exist multiple methods for synthesizing GelMA. Even though the investigation is made to improve these methods, they all follow the general process of Van Den Bulcke [14]. The procedure consists of reacting gelatin with methacrylic anhydride in the presence of phosphate buffer (pH 7.5) at 50 °C. This results in modified gelatin (GelMA) prepolymer. For the preparation of the hydrogel, crosslinking of the gelatin methacrylamide must occur in an aqueous medium in the presence of a water-soluble photoinitiator [11]. The physicochemical properties of the synthesized GelMA depend on two main factors: the level of methacrylation and the source of gelatin. GelMA’s gelatin is usually obtained from mammalian sources like porcine or bovine skin. However, the skin of these animals is restricted by several factors. Like some major religions, around 40% of the population worldwide follows one of these religions. An alternative is the use of marine sources such as fish skin, which can be found as a waste product, and is not restricted by religions [15]. The first synthesis of GelMA was reported in 2000 by Van Den Bulcke et al. [11]. Since then, the publications on GelMA have increased. The rate of studies about GelMA for bone tissue engineering can also be seen with a search on the database of Scopus using the keywords gelatin PRE/1 methacryloyl and another search using the exact keywords and adding the keywords bone and tissue. The results of this research made on the 27th of May 2022 can be presented in a graph (Figure 1).

Hybrid hydrogel systems can be formed by mixing GelMA with nanomaterials, such as graphene oxide and carbon nanotubes, for specific biological applications [16]. Nanomaterial refers to the material whose scale reaches the nanoscale in every direction. Once the material is in the nanoscale, its properties differ significantly from those of its conventional form [17]. According to their functionality, different nanomaterials can be categorized into polymers, metals, ceramics, and composites, where they can replace and/or restore specific biological aspects [18]. In the biomedical field, certain nanomaterials can help to repair damaged body tissue and organs, readjusting body functions. Some nanomaterials have antiviral, anticancer, antioxidant, and antidiabetic activity such as selenium nanoparticles, therefore, they have been used for tackling SARS-CoV-2 [19]. Nanomaterials and nanostructures can also be used as antimicrobial surfaces, solving critical issues in implantable medical devices like the appearance of biofilms, which can lead to infectious diseases or even the death of the patient [20].

**Figure 2:**
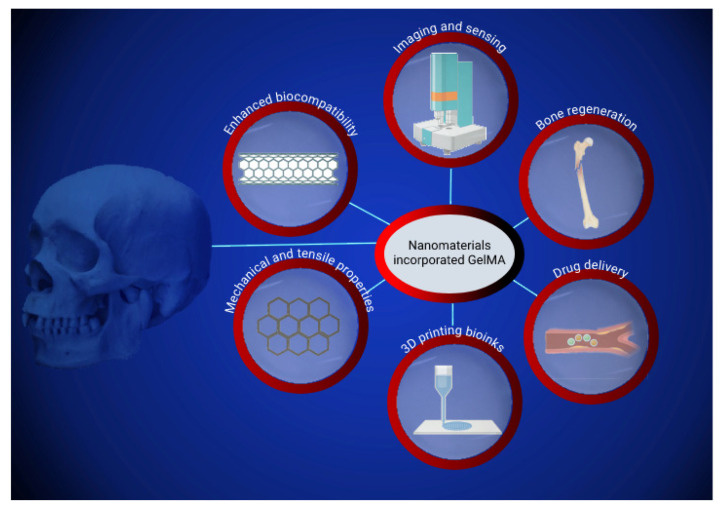
Schematic representation of nanoparticles embedded GelMA composites and their applications.

Recent BTE developments have focused on the 3D printing of nanomaterials used to fabricate custom scaffolds, depending on their application in the body [21]. Scaffold is a type of implant that can be used for drug delivery, overcoming the limitations of conventional drug delivery systems. Aside from a controlled release, scaffolds can be used for the treatment, diagnosis, and regenerative therapy of diseases. The best option to explore these advantages is to use a biodegradable scaffold, for example, GelMA, because it does not require surgery to be removed from the body [22]. The microstructure of the skeletal muscle tissue allows bones to have a strong structure and to perform the needed functions in our body. This allows the manipulation of GelMA to form a similar arrangement to the highly aligned myotubes that form the microstructure of bones [23]. The gel manipulation, the macromolecular characteristics, and the polymer network structures are changed. The current interest of the scientific community is to enhance the mechanical and adhesive properties of such gels [24]. This review aims to present recent applications of different GelMA combinations with nanomaterials in Bone Tissue Engineering. Specifically, the review focus on the recent progress of nanomaterials-incorporated GelMA composites and their physicochemical characteristics and biocompatibility for bone regeneration, drug delivery bone reconstruction, 3D-printing bone scaffolds, and bone-related bioimaging/biosensing (Figure 2).

## 2. Nanoparticle-Incorporated GelMA Nanocomposites

Due to their unique structures and properties, 3D hydrogels and nanoparticles have shown a very high potential for medical and diagnostic applications. The expeditious development of biomedicine increased the demand for multifunctional hydrogels with enhanced mechanical properties [25]. Table 1 summarizes the latest advances of nanoparticles with GelMA to solve the limitations of other GelMA hydrogels. The information in the table is explained in more detail in Section 2.1, Section 2.2, Section 2.3 and Section 2.4, including restrictions or further studies that could be made.

### 2.1. Biocompatibility and Physicochemical Characteristics of Nanoparticles Embedded GelMA Composites

Alcala-Orozco et al., synthesized strontium nanoparticles (Sr-NPs) that created star-like structures via multistage self-assembly. These nanoparticles were further incorporated into a GelMA solution via short vortex spinning, forming a nanocomposite bio-ink (Sr-GelMA) of Sr nanoparticles and low concentration (5 *w*/*v*%) GelMA. The Sr-GelMA nanocomposite targets the current physical and biological limitations of available bio-inks by modulating rheological properties. Sr nanoparticles have been demonstrated to have good biocompatibility; therefore, they have been used before for bone regeneration. The incorporation of Sr to GelMA did not affect the crosslinking reaction. The Sr-GelMA nanocomposite preserved its physical properties, such as pore size and swelling ratio, compared to GelMA alone. However, there was a significant increase in viscosity that enhanced printability. With this bio-ink, 3D hydrogel discs were printed via extrusion. Tests were made in these scaffolds, demonstrating the activity of osteogenic differentiation of human mesenchymal stromal cells (hMSCs) and mineral deposition. Further studies could test the influence of Sr-GelMA nanocomposite in other cell types, such as endothelial cells, chondrocytes, and osteoclasts [26].

In another study, GelMA nanocomposites made of synthetic bones were developed to treat large defect bones. They are made of compounds like hydroxyapatite (HA) and β-tricalcium phosphate (β-TCP). Even though these compounds have favorable characteristics to improve bone function, they have limitations. For example, after the GelMA with HA hydrogels has degraded, HA remains in place, so it is not entirely replaced by new bone. In the case of β-TCP/GelMA hydrogels, complete bone regeneration occurs, but β-TCP is easily degraded in the body. To target these disadvantages, in 2021 a GelMA hydrogel composite was made with biphasic calcium phosphate nanoparticles (BCP-NPs). The mixture consists of HA and β-TCP at (60/40 wt%). The final hydrogel has stability against biodegradation. The use of BCP-NPs improved cell differentiation and improved the mechanical properties of the hydrogel [27]. Previously, biphasic calcium phosphate nanoparticles-loaded hyaluronic acid/gelatin as well as chitosan/gelatin hydrogels have been used for BTE [38,39]. Further research could compare in vitro and in vivo the performance of these three different hydrogels with BCP-NPs. In the same year, Alcala-Orozco et al., fabricated a bio-ink consisting of GelMA, magnesium hydroxide nanoparticles (Mg), and polycaprolactone (PCL). This biological ink was aimed to have strong physical stability as well as natural functionality. The results demonstrated that the Mg-PCL degraded faster than standard PCL, a positive outcome for in vivo implant degradation and bone regeneration. Another good result was that the combination of Mg-PCL enhanced osteogenic differentiation. This regenerative bone scaffold is promising for skeletal tissue regeneration [28]. Additionally, in 2019, Cidonio et al., investigated the benefits of incorporating nanoclay in the GelMA bio-ink. The study focused on including laponite (LPN) nanoparticles, and the results displayed an enhancement in shape fidelity retention. With the LPN-GelMA bio-ink, the human bone marrow stromal cells proliferated with a significant increase in cell number over 21 days compared to GelMA alone. The bio-ink was implanted ex vivo in a chick chorioallantoic membrane (CAM) model to test this gel. The outcome of this test was that the bio-ink constructs could integrate correctly in the vascular chick embryo after 7 days of incubation. The overall results showed the potential application of the cell-laden hydrogel in hard and soft tissue reparation, but further research is needed [29]. Photothermal therapy (PTT) has excellent potential as a bone regeneration method.

**Figure 3:**
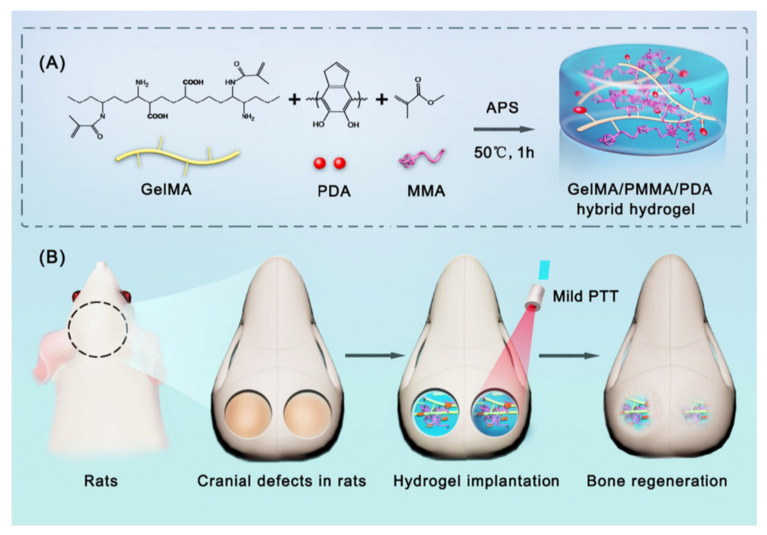
(**A**) Schematical representation of GelMA/PMMA/PDA hydrogel synthesis. (**B**) Illustration of GelMA/PMMA/PDA with mild PTT to regenerate cranial defects in rats. Retrieved from [30]. Reproduced from [30] with permission from Elsevier, copyright©2022.

PTT is based on using light absorbers that create heat from light energy. Polydopamine nanoparticles (PDA) can be used as photothermal agents. They have advantages like good biocompatibility, mild photothermal effect, and easy preparation. PDA can be found in various morphologies. However, in the study of Wu et al., in 2022, spherical PDA was used for the synthesis of a GelMA/poly(methyl methacrylate)/polydopamine nanoparticles hydrogel (GelMA/PMMA/PDA) (Figure 3). This composite was made via free-radical polymerization. poly(methyl methacrylate) (PMMA) was used as an additive to improve mechanical properties and stabilize the structure. Biocompatibility tests were made in vivo. The effects of GelMA/PMMA/PDA hydrogel with mild PTT were tested with a rat cranial defect model. In vitro studies showed that the hydrogel has good biocompatibility, induces osteogenic differentiation, and has an excellent photothermal effect. The hydrogel reached 44.1 °C, and the temperature suitable for bone repair is between 40 °C to 43 °C. In vivo studies showed that the hydrogel maintained a stable structure for the first four weeks and started degradation after the four weeks. It was concluded that the hydrogel with PTT promotes the regeneration of bone defects. Further research is still needed to understand the mechanism of bone repair by mild photothermal hydrogels [30].

### 2.2. Nanoparticle Incorporated GelMA-Based Drug Delivery System for Bone Regeneration

Another type of nanoparticle is soft nanoparticles like nanoliposomes. Nanoliposomes can be included in a GelMA matrix to form a nano-functionalized platform. Elkhoury et al., 2020 synthesized nanoliposomes with salmon lecithin and loaded them with naringin. This compound can induce stem cells’ osteodifferentiation. However, it requires an extensive metabolism. GelMA hydrogels can release drugs in a few hours due to their big pores. Nevertheless, if the drugs are loaded into nanoliposomes, the release can be controlled and prolonged for several days. In the study, when de-loaded nanoliposomes were incorporated into the GelMA matrix, a controlled drug release and high encapsulation efficiency were obtained. Additionally, there were no signals of cytotoxicity toward human mesenchymal/stromal stem cells. The mechanical and rheological properties of GelMA improved and the swelling ratio and hydrophilic character decreased. GelMA hydrogel with naringin-loaded nanoliposomes can be a promising bio-ink. Further studies will focus on the optimization of the nanocomposite hydrogel as a bio-ink and its osteodifferentiation ability in 2D and 3D cell cultures [31]. Mesoporous silica nanospheres (MSNs) enhance the osteogenic potential of hydrogels and can alter their mechanical properties. The mineralization of bone marrow stromal cells can be stimulated with MSNs. They have multiple characteristics that help them function as carriers of bioactivity, such as large surface area, pore volume, and good biocompatibility. In the work of Qu et al. in 2021, MSNs were carboxylated, resulting in MSNs-COOH. Then, these nanospheres were loaded with metformin (MF), a drug that supports bone formation used in type 2 diabetes, resulting in Mf-MSNs-COOH nanospheres. One of the main challenges with MF is that it can rapidly dilute if there is no optimal storage and drug release control. The loaded nanospheres were added to a GelMA solution to address this problem, and photocrosslinking was used to make the hybrid hydrogel of MF-MSNs-COOH/GelMA. The incorporation of MSNs-COOH obtained the highest compressive modulus and swelling ratios to GelMA in 1.5 mg/mL. The cytocompatibility was examined with human exfoliated deciduous teeth (SHEDS). It was concluded that MF-MSNs-COOH/GelMA is a promising hydrogel for injectable bone regeneration therapy, focused on craniomaxillofacial applications. Further studies are still required to find the adequate concentration of Metformin for MSNs in GelMA [32].

Another study of nanocomposites as bio-inks was made by Tavares et al. in 2021. In this study, a multi-bioactive nanocomposite bio-ink was made with GelMA, human bone marrow-derived mesenchymal stem cells (hBM-MSC), and functionalized mesoporous silica nanoparticles (MSN). As MSN has a mesoporous structure, it can act as a nanocarrier. Dexamethasone (Dex), calcium (Ca), and phosphate (P) ions were incorporated into the MSN. The final functionalized nanoparticles (MSNCaPDex) can promote stem cell osteogenic differentiation with just one administration. The manufactured 3D bio-ink was composed of the bone matrix’s major significant organic (GelMA) and inorganic (MSNCaPDex) components. It showed promising results like the autonomous promotion of pro-osteogenic differentiation without adding another osteogenic supplementation. This bio-ink is an excellent option for 3D extrusion bioprinting. Further studies should focus on the living constructs after implantation in bone defects to test if the bioactive and pro-osteogenic capabilities are maintained [33].

### 2.3. Nanoparticles Incorporated GelMA Composites for Biomimetic Hydrogel Periosteum

**Figure 4:**
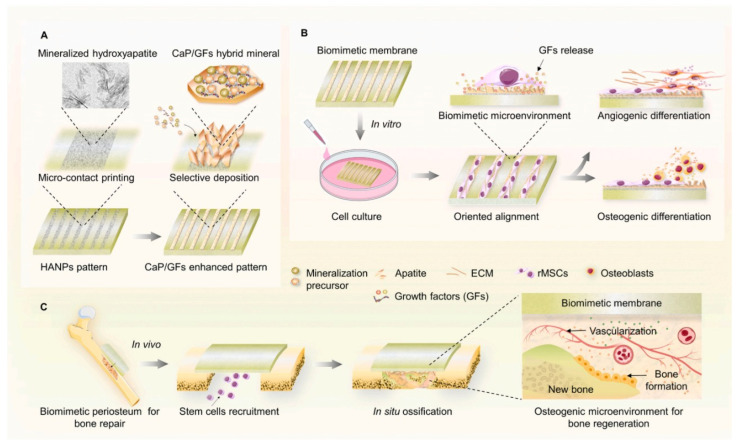
Schematic illustration of the synthesis of a mimicking periosteum membrane with nanoparticles. (**A**) Use of apatite selective deposition for the generation of a biomimetic membrane with mineralized micropattern. Illustration for the potential effects of the periosteum-mimetic membrane with the mineralized pattern on (**B**) stem cell differentiation in vitro, and (**C**) vascularized osteogenesis in vivo. Retrieved from [41]. Reproduced from [41] with permission from Elsevier, copyright©2021.

The periosteum is a membrane located at the outer surface of bones. It can work as a source of growth factor due to its rich cellular composition, like differentiated osteoprogenitor cells, mesenchymal cells, and osteoblasts. When there is a high-energy trauma, bone defects can occur, and the periosteum can be damaged. This membrane provides blood to the bone, in case of damage, bone healing will be delayed [40]. Several artificial periostea have been made (Figure 4), but most of them only focus on the osteogenic activity, overlooking angiogenesis activity [34]. In 2020 Liu et al., made a mimicking periosteum. The hybrid biomimetic periosteum was made with electrospinning by using organic and inorganic materials. The inorganic part was composed of calcium phosphate nanoparticles (CaPs). The emulsion method synthesized these nanoparticles with a diameter of 50.7 ± 2.3 nm. CaPs were further incorporated into GelMA, the organic component, with electrospinning. Using inorganic components helps in the regeneration of hard tissue, improves the stiffness and mechanical strength of the system, and enhances osteogenic differentiation. CaPS also provide bioactivity to the system since they can work as drug carriers, and their released ions are essential in the process of bone formation. The final hybrid hydrogel fibers showed controlled ions release for over 10 days, mineralization appeared on the surface of the fibers after coincubation with simulated body fluid (SBF), and osteogenesis and angiogenesis capabilities were verified with human umbilical vein endothelial and MC3T3-E1 cells [34].

Another research work that focuses on mimicking periosteum with promoting angiogenesis and osteogenesis was made by Yang et al. They made a periosteum mimicking bone aid (PMBA) with electrospinning and then photocrosslinking three components: GelMA, L-arginine-based unsaturated poly (ester amide) (Arg-UPEA), and methacrylated hydroxyapatite nanoparticles (nHAMA). Arg-UPEA is added to increase the adhesion strength of the nanofibrous hydrogel membranes. The function of nHAMA is to improve mechanical properties and provide calcium ions (Ca^2+^). In the company of sufficient NO production, the degradation products obtained (Ca^2+^ and L-arginine) will trigger a series of processes that eventually end in osteogenic-angiogenic activity. The final PMBA showed biocompatibility for its use in surgical procedures, strong tissue adhesion, and a comparable elastic module to natural periosteum [35].

### 2.4. Nanoparticles-Incorporated GelMA Scaffolds for Bioimaging

In the work of Celikkin et al. in 2019, gold nanoparticles (Au-NPs) have been used in GelMA as a contrast agent. BTE is progressing toward clinical applications, hence the importance of imaging methods, such as computed tomography (CT), to evaluate the results of tissue-engineered constructs. The X-ray attenuation shows the material’s link to its density and atomic number. Therefore, mineralized tissues will have high contrast while soft tissues, composed mainly of water, have low contrast. Hydrogels, like GelMA, have a weak CT signal. By incorporating Au nanoparticles into GelMA pre-polymer solution, 3D GelMA-AuNPs scaffolds were obtained. Different concentrations of Au nanoparticles were used, and the best mechanical, enhanced radiopacity, and cytocompatibility performance, occurred with 0.16 mM 60 nm Au nanoparticles. With the incorporation of Au nanoparticles, the attenuation was increased. The scaffolds had osteogenic features and good cytocompatibility. These results demonstrate that Au nanoparticles can help image GelMA through CT, not only the newly formed bone. Further studies could include the effect of having Au-NPs in the composites discussed in Section 2.1, Section 2.2 and Section 2.3 [36].

**Figure 5:**
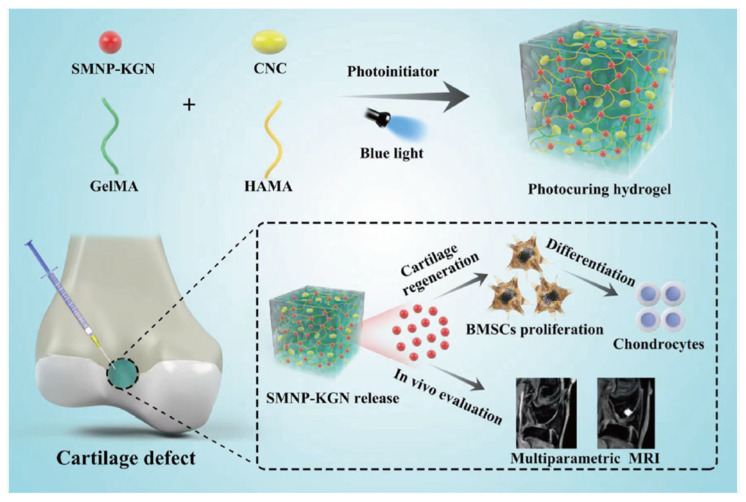
Preparation and functionalization of kartogenin-loaded and SMNP-labeled multifunctional hydrogel scaffold for cartilage regeneration. Retrieved from [37]. Reprinted from [37] with permission of John Wiley & Sons, copyright©2017.

Some studies have been made attempting to promote chondrocyte differentiation using kartogenin (KGN). Still, the results showed that KGN by itself will present a loss of KGN or absorption into the circulatory system. Another problem with the traditional surgical procedures for articular cartilage remains unsatisfactory. Some studies have tried to find noninvasive methods to monitor the functionalization and degradation of tissue engineering scaffolds in vivo. One example is fluorescence imaging, but due to the poor resolution of the image, further application of this method is limited [42]. For this reason, Chen et al., designed a blended hydrogel scaffold system loaded with synthetic melanin nanoparticles (SMNP) and KGN for theragnostic purposes. The results demonstrated that this SMNP-KGN/Gel had strong mechanical properties, thermal stability, and enhanced magnetic resonance imaging (MRI). The release of the drug KGN could make bone-derived mesenchymal stem cells proliferate and differentiate in vivo (Figure 5) [37].

## 3. Nanotubes-Incorporated GelMA Composites

As a novel method, the use of three-dimensional scaffolds contributes to treatments for cartilage and bone injuries yet scaffolds alone cannot be compared to the complex morphology of these both. However, emerging nanocomposites like carbon nanotubes (CNTs) show outstanding physicochemical properties that aid in reinforcing bone and cartilage scaffolds using a bioprinting technique in 3D. CNTs showed different advantages, such as superior mechanical properties with tensile strength, and the surface mimics collagen fibers making them ideal for 3D support of cell proliferation, differentiation, and communication (which are all important for angiogenesis and vascularization). It also increases the biocompatibility of the scaffold, thanks to the interaction with extracellular proteins and higher cell interaction. Some major drawbacks are the scaffold production that accommodates the fibers with an effective structure and porosity. Yet, the lack of long-term research is a significant drawback for the scaffolds to be used in the market since the scaffolds may be promising. However, they are not enough to mimic the native tissue. Additionally, 3D bioprinting is still a novel technique and further studies need to be carried out [42].

Recently, many biomaterials have been created for bone regeneration, yet this process can be lagged or interrupted by bacterial infection and immune response. However, to defeat these situations, QianminOu and companions fabricated a nAg/HNTs/GelMA hybrid hydrogel. The nAg/HNTs/GelMA hybrid hydrogel was made to accelerate bone regeneration with osteo-immunomodulatory and antibacterial activities. This biomaterial demonstrated good biocompatibility with human periodontal ligament stem cells (hPDLSCs) and macrophages, regulation of cytokines released by macrophages, and showed a controlled osteoimmune microenvironment by eliminating bacterial infection. The nAg/HNTs/GelMA hydrogel shows a novel approach to treating infected bone defects [43]. Hydrogels lack the mechanical strength to mimic tissue or even the biological extracellular matrix (ECM). If a hydrogel can mimic ECM, there would be a higher possibility of cell signaling and better biocompatibility. Therefore, Su Ryon Shin and colleagues presented a reinforced Carbon Nanotubes–gelatin methacrylate (GelMA) hybrid to mimic the native tissue-like structure and function with a three-dimensional design. These carbon nanotubes (CNTs) aim for enhanced mechanical strength, taking care not to shrink their porosity or hinder cell growth. The CNT–GelMA hybrids had patternable photo properties, which can be used for microscale structures without unpleasant processes. NIH-3T3 cells and human mesenchymal stem cells increased after the encapsulation in CNT–GelMA hybrid microgels, varying the number of CNTs, depending on the mechanical properties desired. These biomaterials demonstrated a good use for tissue engineering.

Furthermore, the incorporation of CNTs can also be used to produce 3D biomimetic tissue-like structures [44]. Nanotubes have been widely applied for bone regeneration treatments, some of them are carbon nanotubes and titanium dioxide nanotubes. Yet, some limitations occurred because of their degradability and composition, therefore, it is expected that nanotubes with good biocompatibility, functionality, and structure can aid in bone regeneration. Keqing Huang and his team presented halloysite nanotubes (HNTs) incorporated hydrogel produced by a photopolymerization method and gelatin methacrylate (GelMA). Halloysite is a naturally occurring aluminosilicate nano clay. With the implementation of HNTs, the mechanical strength was enhanced, it demonstrated cytocompatibility in vitro, and it demonstrated overexpression of osteogenic differentiation-related genes, and the associated protein of human dental pulp stem cells (hDPSCs).

**Figure 6:**
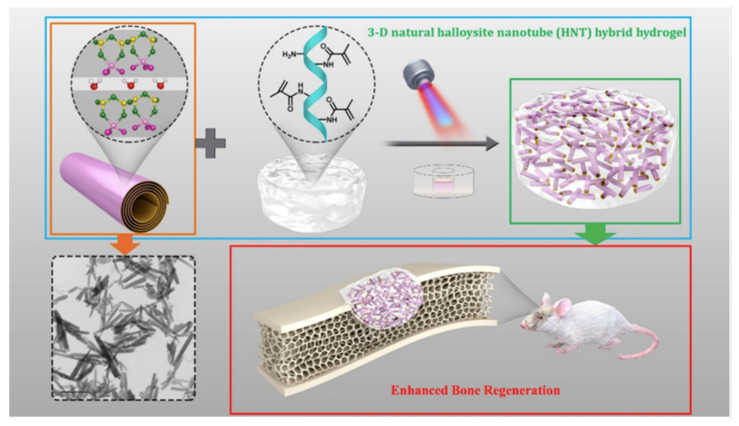
Halloysite scaffold with nanotubes and GelMa for bone tissue engineering in rats. Retrieved from [45]. Reproduced from [45] with permission from ACS, copyright©2019.

All these previous factors smoothed the path for bone regeneration in a clinical trial using rats with calvarial defects, showing a favorable alternative for bone regeneration and bone tissue engineering (Figure 6) [45].

(Ti)-based implantations in the orthopedic field typically produce non-desired osseointegration and bacterial-associated infections. TiO_2_ nanotubes (TNT) loaded with bone morphogenetic protein 2 (BMP2) were made together with MA-modified gelatin (GelMA) and N-Cl modification poly (N, N′-methylene bis(acrylamide)) (PMAA-Cl) to tackle these circumstances. Previously, it was done through a layer-by-layer (LBL technology) self-assembly technique. These nanotubes exhibited antibacterial properties against Escherichia coli (*E. coli*) adhesion and development and Staphylococcus aureus (*S. aureus*). On the other hand, studies revealed that the TiO_2_ nanotubes encouraged osteoblasts’ adhesion, proliferation, and differentiation. The previous means that the LBL samples have outstanding biocompatibility with osteoblasts and antibacterial effects. These substrates introduced novel titanium surface modifications in orthopedic implants [46].

When using hydrogel systems, it is expected to be able to boost the regenerative capacity of endogenous progenitor cells via the localized presentation of therapeutics; all these under inflammatory conditions can be promising for complex tissue regeneration. In this case, Ester A. F. Bordini and his colleagues, used dexamethasone (DEX), a multi-effect drug with anti-inflammatory and mineralizing capacity, and halloysite clay nanotubes (HNTs) to fabricate an injectable multifunctional drug delivery system based on photo-cross-linkable GelMA hydrogel. Several GelMA formulations with different amounts of DEX-loaded nanotubes were analyzed and demonstrated an enhanced mechanical strength of GelMA, with no alterations in its degradation ratio, swelling ratio, or porous anatomy of gel. In vivo data showed that DEX-loaded nanotube-modified GelMA (5.0% HNT/DEX 10%) enhanced bone formation after 6 weeks, with a minimum localized inflammatory response after 7 days. This demonstrated that DEX-loaded nanotube-modified GelMA compared to DEX-free formulations shows a better behavior of tissue regeneration under inflammatory conditions [47]. With the use of dielectrophoresis (DEP), carbon nanotubes were vertically aligned in GelMA hydrogels in a very efficient style. GelMA-aligned CNT hydrogels demonstrated anisotropic electrical conductivity and superior mechanical properties compared to those hydrogels where CNTs are distributed in a randomized way. Furthermore, the GelMA-aligned CNT hydrogels revealed a development of functional myofibers when skeletal muscle cells were added to it and increased myogenic gene and protein expression with electrical stimulation, compared to those gels with no alignment. In addition, with electrical and mechanical properties, these hydrogels can captivate new applications in the biomedical field [48].

## 4. Graphene-Incorporated GelMA Scaffolds

Another type of nanostructured material that provides remarkable properties for BTE is graphene, together with its derivatives, such as graphene oxide (GO) and reduced graphene oxide (rGO). The strength of the bonds between carbons gives these nanomaterials enhanced electrical, mechanical, optical, sensing, electron and heat transfer, biocompatibility, and drug-loading properties [13,49]. Moreover, the surface and peripheral structure of these materials also give them the ability to change their interactions within bone tissue environments. Currently, available literature containing GelMA-graphene with studied applications directly in BTE is limited, nevertheless, the functionalization of hydrogels (including GelMA) with this nanostructured material has been a point of interest in recent investigations. An example of functionalized hydrogels that could be useful in bone regeneration processes is the work of Jiao et al. [50] where bare gelatin was crosslinked with reduced graphene oxide created, which enhanced the adhesion and proliferation of bone marrow stromal cells (BMSCs). Another type of hydrogel that was functionalized with graphene oxide is the polyacrylamide-sodium carboxyl methylcellulose (PMC), together with cellulose nanocrystals. This mentioned work was published by Kumar and colleagues [51], and the hydrogel showed enhanced and exceptional mechanical and structural properties. GelMA hydrogels reinforced with graphene derivatives may also be found for tissue reparation apart from bone tissue. Both the teams of Rehman [52] and Shin [53] synthesized GelMA-rGO, having applications for chronic wound healing of diabetes patients and cardiac tissue engineering, respectively. Apart from the reports mentioned, some of the studies that show direct applications of GelMA functionalized with graphene and its derivatives in BTE are presented below (Table 2). 

**Figure 7:**
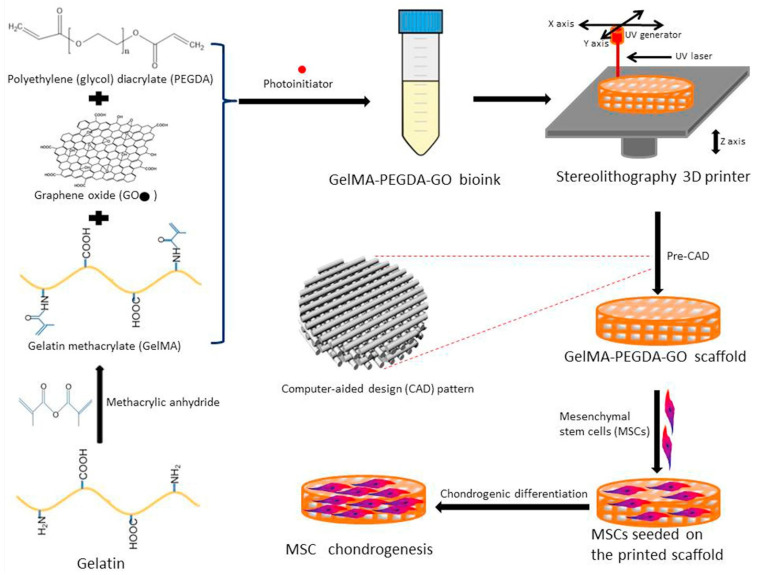
3D printed GO scaffolds for promoting chondrogenic differentiation. Retrieved from [55]. Reproduced from [55] with permission from Elsevier, copyright©2017.

Chondrocyte cells have an important role in producing and maintaining the cartilaginous matrix. Nevertheless, its weak differentiation capacity limits the capability of these embedded cells in ECM for repairing and generating articular cartilage. Alternative cell sources have been proven to enhance the chondrogenic capacity in cartilaginous tissue, such as mesenchymal stem cells (MSCs). To promote the chondrogenic differentiation of hMSCs, in the 2017 team Zhou synthesized GelMA-PEGDA-GO bioink via 3D printing, using the stereolithography (SL) technique (Figure 7) [55]. PEGDA was used to improve the printability of the GelMA bio-ink. At the same time, GO carbon domains and hydrophilic functional groups were expected to enhance the biochemical function of the GelMA-PEGDA printing ink. For the different tests and characterizations, GelMA-PEGDA scaffolds were printed with and without GO. After the SL process, GO’s different concentrations did not influence the scaffold’s structural fabrication or compressive modulus. After choosing an adequate proportion of GelMA-PEGDA in the scaffold based on the proliferation of MSCs, incorporating different amounts of GO proved to promote MSC adhesion, growth, and differentiation in all the tested concentrations. The protein affinity of the scaffolds was measured using BSA as a model protein. While incorporating GO, the adsorption profiles were 763% higher than the GelMA-PEGDA scaffolds after 24 h. The incorporation of GO also dramatically improved the collagen II synthesis, GAG secretion, and total collagen level in the ECM [55].

The significant increase in the differentiation of hMSCs might lead to the idea that hybrid hydrogels are adequate solutions for healing and regenerating bone cartilage at a cellular level.

The team of Mamaghami [56] found different results from the ones obtained by Zhou and colleagues, inferring that the incorporation of GO in GelMA hybrid gels indeed changed the compressive modulus of the hydrogels, apart from other mechanical properties. In 2018, Mamaghami presented studied the mechanical properties and swelling behavior of an interpenetrating network (IPN) hydrogel containing GelMA, PEGDA, and GO nanoparticles. Although the presented study did not report any cytotoxicity, biocompatibility, or osteogenesis test, the conducted analysis presented an inference on how the hydrogel could work in a tissue environment, taking a mechanical scope. GelMA/PEGDA hydrogel containing GO nanoparticles was synthesized via photopolymerization reactions, followed by pressure tests to determine its mechanical properties. The concentrations of the three components inside the IPN hydrogel were varied, so the compressive modulus could be compared concerning the polymers and GO nanoparticle concentrations. The GelMA hydrogel elastic modulus increased noticeably by increasing the concentration of GelMA. After this, the incorporation of 10 wt% PEGDA to 5 wt% GelMA raised the compression modulus from 8.2 ± 2.1 kPa to 192.1 ± 22.9 kPa. The addition of 0.8 mg/mL GO nanoparticles followed the same trend, elevating the elastic modulus of GelMA hydrogel and IPN hydrogel (5 wt% GelMA and 5% PEGDA) by almost 50% in both cases [56]. This mechanical enhancement could cover the limited strength of the hydrogel while applying it to specifically hard bone tissues. The swelling ratio of the synthesized hydrogels was measured after 24 h of water absorption. The trend seen in mechanical properties was found to be inverse: by increasing GelMA concentrations, the swelling ratio of the bare GelMA hydrogel decreased in a significant matter. The same happened while adding 10 wt% PEGDA to 5 wt% GelMA, reducing the swelling ratio from 18.2 ± 3.7% to 2.4 ± 0.4%. The same was seen by adding GO (0.8 mg/mL) nanoparticles in both the GelMA hydrogel and the IPN hydrogel, decreasing the swelling ratio from 18.2 ± 3.7% to 16.0 ± 1.19% and from 4.7 ± 0.7% to 3.7 ± 0.7%, respectively. The density of the hydrogel increased by adding PEGDA and GO nanoparticles, reducing the available space for the hydrophilic activity of the hydrogel [56].

**Figure 8:**
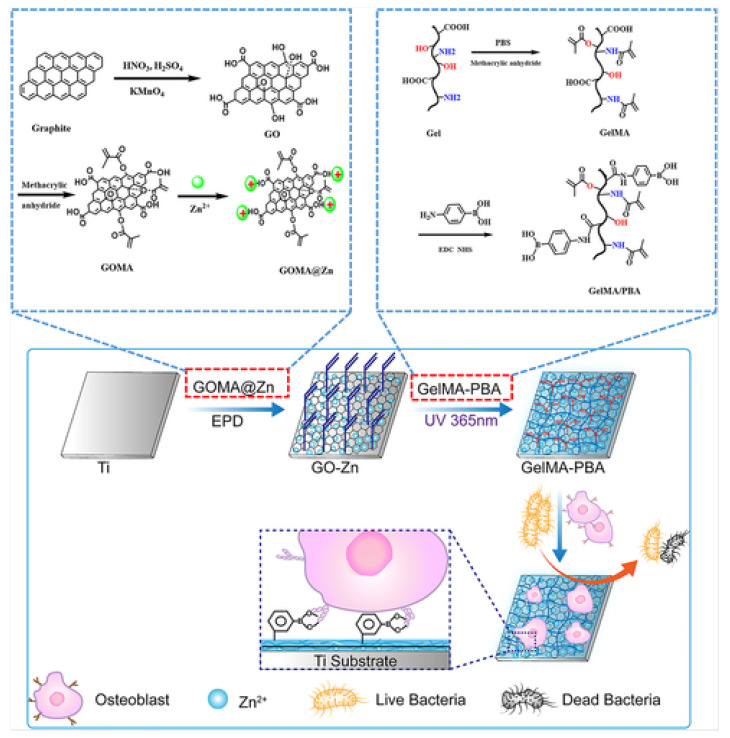
The schematic illustration of the fabrication process of the GO-Zn/GelMA-PBA system and its osteogenic and antibacterial properties. Retrieved from [57]. Reproduced from [57] with permission from Wiley, copyright©2019.

To test the mentioned mechanical and biological properties of GelMA-GO hydrogels, the team of Tao [57] analyzed osteogenic activity and bacterial adhesion of hybrid metallic-organic implants. Metallic materials and their alloys have been widely used in orthopedic and dental implants, such as titanium (Ti) and its alloys, due to their mechanical properties and good compatibility. Nevertheless, in BTE, Ti cannot integrate into the cartilage because of its bioinert surface. Another metallic material with wide applications in the biomedical field is Zinc (Zn). Zn ions enhance the osteogenic differentiation of osteoblasts, improving ALP activity, ECM mineralization, and genes or protein expression. These last ones are involved in osteogenesis processes. Taking this into account, the team proposed and synthesized Ti substrates with different layers, such as GO-Zn and GelMA-PBA coatings, seeking a Zn source and enhancing osteoblasts proliferation. Additionally, GelMA-PBA could work as an antibacterial agent and enhance osteogenic differentiation. In experimental procedures, three groups of substrates were synthesized and tested: GO-Zn, GO-Zn/GelMA, and GO-Zn/GelMA-PBA, together with native Ti substrates (Figure 8) [57].

**Figure 9:**
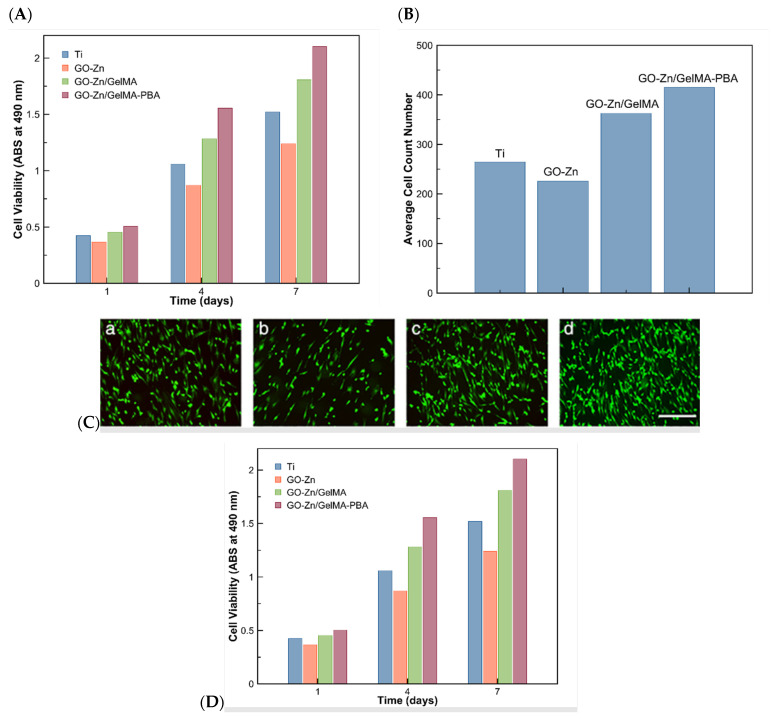
(**A**) BSA adsorption onto different Ti substrates after culture for 2 and 4 h, (**B**) quantitative measurements of the number of adhered cells on various samples after co-culture for 2 days, (**C**) live/dead staining of osteoblasts grown onto different substrates after culture for 48 h: bare Ti (**a**), GO-Zn (**b**), GO-Zn/GelMA (**c**), and GO-Zn/GelMA-PBA (**d**), respectively. Scale bar: 100 μm, (**D**) osteoblasts proliferation on different substrates after culture for 1, 4, and 7 days. Retrieved from [57]. Reproduced from [57] with permission from Wiley, copyright©2019.

For testing protein absorption capacity, BSA experiments were carried out. After 4 h, tuned substrates showed more BSA absorption than bare Ti samples (Figure 9A). Between the synthesized substrates, GO-Zn/GelMA-PBA was the one showing more protein absorption. The number of adhered cells was significantly lower on the GO-Zn substrate than in the bare Ti group (Figure 9B); however, this number was outscored by the GO-Zn/GelMA and GO-Zn/GelMA-PBA groups samples (almost all cells on GO-Zn/GelMA-PBA substrates remained alive). To measure the samples’ cytotoxicity, a live/dead assay was conducted (Figure 9C), where more osteoblasts were spread on GO-Zn/GelMA-PBA samples. Similar trends were observed in the osteoblast proliferation test (Figure 9D). After 4 days, the viability of osteoblasts increased in the following order: GO-Zn, Ti, GO-Zn/GelMA, and GO-Zn/GelMA-PBA [57].

Osteoblastic differentiation was tested with different methods. ALP activity was measured, where the osteoblasts grown on GO-Zn/GelMA-PBA substrates secreted more ALP than the other groups. The same test was carried out on a gene level, using qRT-PCR analysis, where the expression levels of genes involved in bone formation were evaluated. Expression levels of Runx2 in the GO-Zn/GelMA-PBA sample were noticeably higher than in the other groups, which agreed with the expression levels of Osterix, ALP, Col 1, OPN, and OCN [57]. GO-Zn/GelMA and GO-Zn/GelMA-PBA groups also showed more microbial activity. For this evaluation, *P. aeruginosa* (G-bacteria) and *S. aureus* (G + bacteria) were used. GO-Zn/GelMA and GO-Zn/GelMA-PBA reduced their presence by 66.5 and 64.8%, respectively, for *P. aeruginosa* compared to bare Ti substrate. The same trend was observed in the reduction of *S. aureus.* This test was complemented with the plate counting method and agar diffusion essay. After an incubation period, the same cultured bacteria for the previous analysis were accumulated in a large amount on the substrates, followed by GO-Zn/GelMA and GO-Zn/GelMA-PBA. Nevertheless, GO-Zn was the group that showed less bacterial accumulation. This study shows that the biocompatibility, antibacterial properties, and osteogenic activity of bare Ti substrates may be enhanced with crosslinking of ion sources and biofilms, giving the metallic substrate new properties for bone regeneration and osteoblasts differentiation, apart from enhanced mechanical properties [57]. Later in 2021, the effects of GO-hydrogel incorporation in osteogenesis processes were deeper studied by Li and colleagues [58]. It has been reported that silicon (Si) has also enhanced properties in bone metabolism. Therefore, the team tested the osteogenesis of hMSCs, generating 3D-printed scaffolds of GelMA-GO and GelMA-SiGO. Three tests were carried out: the presence of BMP proteins (growth factors in osteogenesis), the inhibition of the osteoinductive potential of the nano-structured scaffolds due to the fact of LDN (BMP antagonist), and the in vivo bone formation of a mouse subcutaneous model. GO and Si-GO-functionalized hydrogels were biocompatible; nanostructured scaffolds did not show any changes in the equilibrium water content (EWC) concerning the GelMA scaffold.

**Figure 10:**
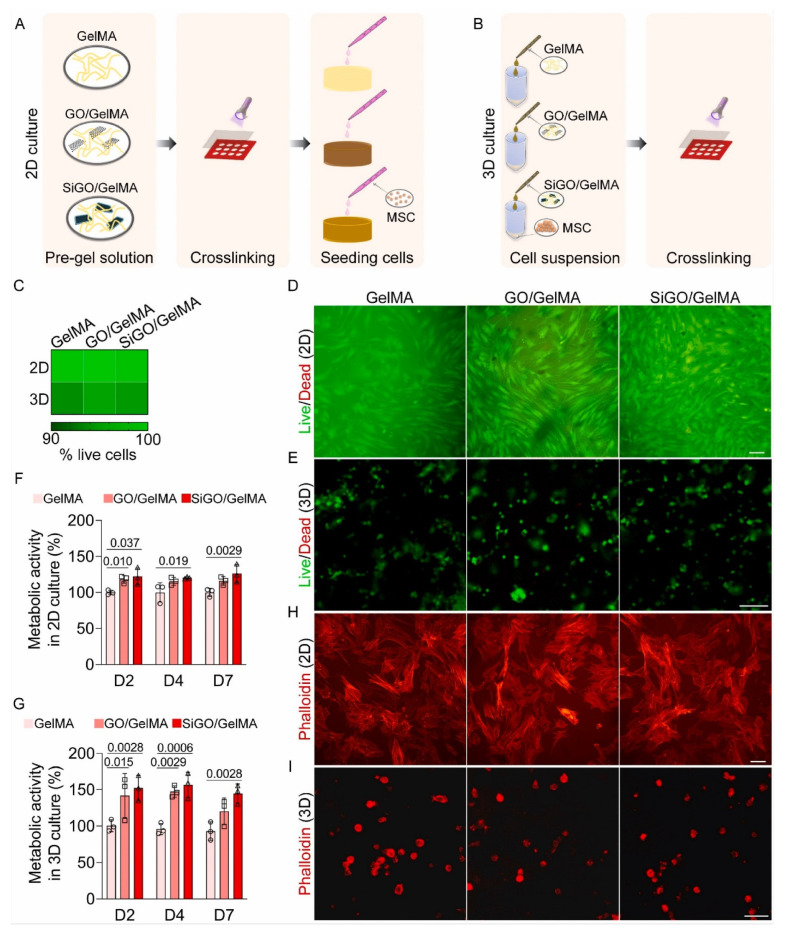
Cytocompatibility assessment of GO- and SiGO-functionalized hydrogels. (**A**,**B**) Schematics of the establishment of (**A**) 2D and (**B**) 3D cultures. (**C**) Heatmap showing the percentage of live cells in 2D and 3D cultures. N = 3. (**D**) Representative LIVE/DEAD assay images. hMSCs were cultured on three types of hydrogels for 4 days. Scale bar = 100 μm. (**E**) Representative LIVE/DEAD images of hMSCs after 4 days of culture within 3D scaffolds. Scale bar = 100 μm. (**F**) hMSCs were seeded on TCP and cultured in the scaffold-conditioned medium. The alamar Blue assay measured metabolic activities on days 2 (D2), D4, and D7 and normalized to the GelMA group. N = 3, one-way ANOVA was carried out. *p* values were labeled. (**G**) Metabolic activity of cells encapsulated in the hydrogel scaffolds normalized to the GelMA group. N = 3, one-way ANOVA was carried out. *p* values were labeled. (**H**) Phalloidin staining of hMSCs after 4 days of 2D culture on the scaffolds in OM. Scale bar = 100 μm. (**I**) Phalloidin staining of hMSCs after 4 days of culture in hydrogels. Scale bar = 50 μm. Schematics Retrieved from [58]. Reproduced from [58] with permission from Elsevier, copyright©2021.

Cultures hMSCs were also live/dead tested on the top (2D) and encapsulated (3D) in the three (Figure 10A,B, respectively). In the three groups, only a few of the cells were dead; nevertheless, more living cells were observed in the nanostructured gels (Figure 10C–E). These tests proved the lack of toxicity in the hydrogels while interacting with hMSCs [58]. Both in 2D and 3D, nanostructured scaffolds showed higher metabolic activity compared to the naïve GelMA (Figure 10F,G). The morphology of the cells was examined by phalloidin staining of cytoskeletal actin filaments. Figure 10H shows the attachment and spreading of cells, while Figure 10I shows round structures.

**Figure 11:**
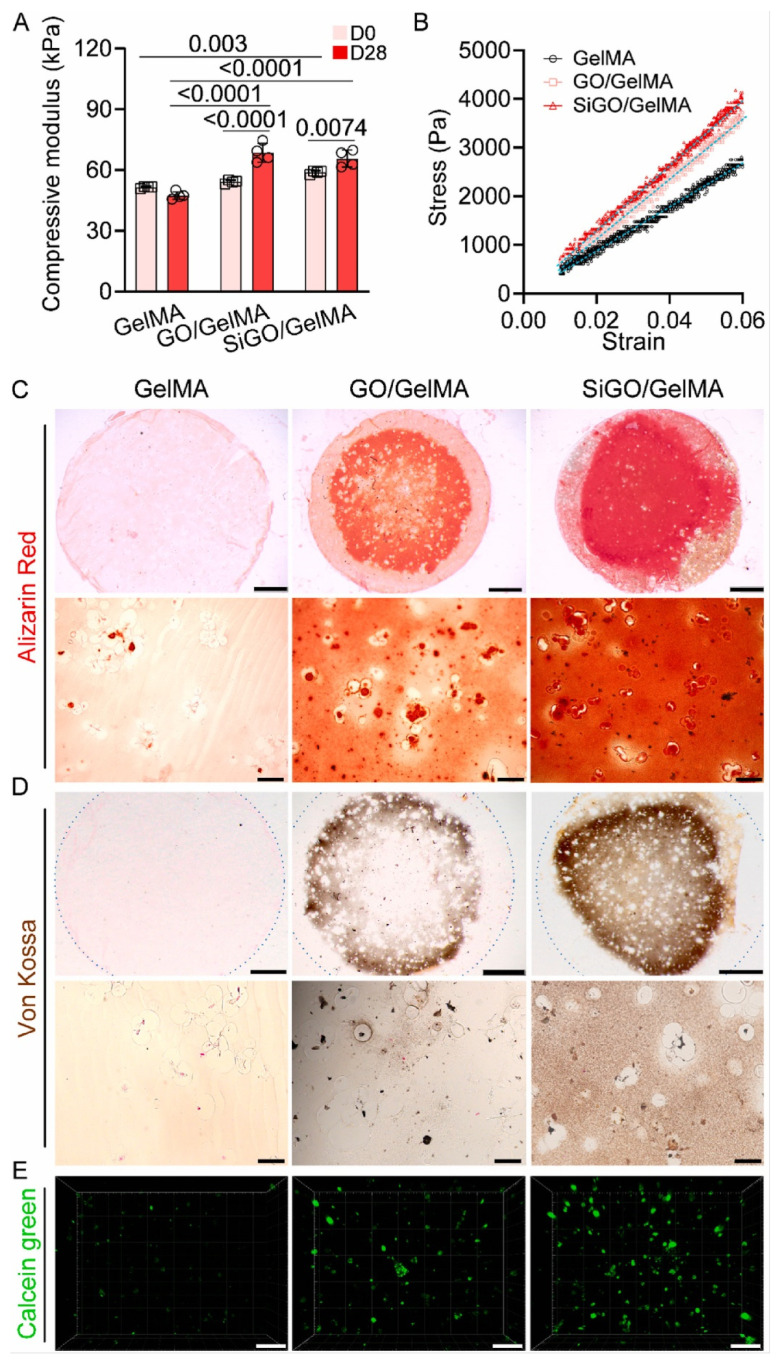
Assessment of mechanical property and mineralization. (**A**) The compressive modulus of cell-laden scaffolds before and after 28 days of culture in OM. N = 4, data analyzed by unpaired *t*-test. *p* values were labeled. (**B**) Representative stress–strain curves of the D28 samples. (**C**,**D**) Alizarin Red staining (**C**) and von Kossa staining (**D**) images show mineralization in the constructs after 28 days of culture in OM. The dotted circles in D depict the outline of the entire section. Scale bars = 1 mm (top row) or 50 μm (bottom row). (**E**) Confocal images of calcein green-stained calcium minerals in the D28 samples. Scale bar = 100 μm. Retrieved from [58]. Reproduced from [58] with permission from Elsevier, copyright©2021.

GO and SiGO hydrogels also showed a higher compressive module than the GelMA scaffold and higher stress levels at similar strains (Figure 11A,B, respectively). The expression of BMP-2 biomarkers was tested with rt-qPCR, and the SiGO-GelMA scaffolds were the ones that increased the expression of the proteins in a higher manner [58]. Alizarin Red, Von Kossa, and calcein staining were used to test a mouse subcutaneous model’s in vivo bone formation (Figure 11C–E). Nanocomposites showed an increase in bone formation only after 4 weeks of implantation, significantly more than the GelMA group [58].

In the biomedical field, in vivo studies can prove how materials like Si and GO are adequate for enhancing the bone regeneration processes, leaving space for more research toward diminishing the time for complete bone healing after trauma. In the same year, Geng and teammates found that the surface charge polarity of BTE materials may change their physicochemical and biological properties in the interactions with hMSCs [59]. Both positive and negatively charged materials have been proven to enhance osteoblastic proliferation, but there is currently no specific rule or direct relationship between surface charge and effects in BTE. The team synthesized three types of graphene quantum dots (GQDs) through a rapid microwave-assisting reaction: positively, negatively, and almost neutrally charged, respectively. The different functional groups that can be added to the GQDs may change their surface charge while keeping surface chemistry, size, and morphology unchanged. Cultured hMSCs were treated with the three types of GQDs. No oxidative stress was observed in any samples, revealing that the different-charged nanomaterials induced no significant cytotoxicity. Out of the three types of GQDs, the negatively charged GQDs group showed enhanced osteogenic differentiation and more calcium mineral nodules, leading to the selection of these GQDs for the hydrogel synthesis using GelMA. GQDs^−^ were linked to GelMA using a UV-light-activated photopolymerization; hydrogel was released in a mouse calvarial defect model for testing the in situ bone regeneration. The brownish GQDs^−^-GelMA hydrogel showed a porous sponge-like structure, making the gel adequate for water absorption. The swelling rate of both GelMA and GQDs^−^-GelMA hydrogels was tested, where the GQDs^−^ incorporation showed an improvement in water absorption concerning the GelMA hydrogel. The degradation of the hydrogel in the bone tissue is crucial for BTE, so the synthesized hydrogel was immersed in an aqueous environment, where after 25 days, the GQDs^−^-GelMA hydrogel decreased to almost 30% of its original mass. Mechanical properties were also enhanced with the incorporation of GQDs^−^ in the GelMA hydrogel, where the compression modulus was slightly increased. The biocompatibility of hydrogel was tested using live/dead staining, and the studies revealed that the adequate proliferation of hMSCs combined with the GQDs-GelMA hydrogel proves the biocompatibility of the nanostructured gel. Osteogenic effects of the synthesized hydrogel were also fair and better concerning the GelMA hydrogel: after 4 weeks, the bone defects of the calvarial mouse model were almost completely healed with the GQDs^−^-GelMA hydrogel, showing better results than the GelMA gel. The study concluded that the biodegradable negatively charged nanomaterial was able to enhance bone tissue regeneration, together with the GelMA hydrogel. This also leads to further studies where the surface charge of hydrogels may be varied, showing different results and applications in the biomedical field [59].

## 5. Challenges and Alternative Approach

Bone regeneration treatments involve surgical intervention, which required a lengthy recovering time. For this reason, new strategies are pursued, including biological, osteoinductive, and hybrid materials [5]. Some treatments proposed in this review including nanoparticle-incorporated GelMA nanocomposites that increase physical stability, bio-logical functionality [26], have a controlled drug release, [31] and allow osteogenesis activity while having angiogenesis capability [35]. However, some of the limitations include the degradation of compounds such as HA and β-TCP when incorporated into the GelMA nanocomposite, this enables a semi-regeneration of the bone tissue. Other treatments included in this review are nanotubes incorporated in GelMA scaffolds. A drawback of these scaffolds is that the printing must be precise to allow a correct and effective structure and porosity, for this reason, the mimicking of native tissue is not achieved [43]. One last treatment shown in the review is graphene-incorporated GelMA scaffolds. Its main challenge is the weak differentiation capacity that limits the capability of the cells in ECM for repairing and generating articular cartilage [55]. Other approaches that could be investigated to promote better bone regeneration focus on mimicking the bone microenvironment. To make this, several nanomaterials can be used such as HA nanowires, nanodiamonds, carbon dots, titanium dioxide nanotubes, MXene nanosheets, bioactive glass, scaffolds (metal-based, bio-ceramic, polymeric, natural or synthetic). More details are provided elsewhere [60]. Another example of an alternative is the use of guided bone regeneration membranes from type I and type III of cattle and pigs [61].

## 6. Conclusions and Future Prospective

Various vital characteristics and preparation methods of GelMA-based composites have been reviewed for bone tissue engineering. GelMA is attained from natural gelatin polymer via a single-step chemical process. The chemical modification of the methacryloyl functional group enables accessible and rapid gelation of GelMA under UV irradiation in the company of photo-initiators. The physicochemical properties of GelMA composites, including mechanical characteristics, degradation rates, porosity, swelling ratio, and gelation, can be readily tuned by initiator concentration, the degree of methacryloyl substitution, and UV light irradiation time. Even after crosslinking and chemical modification, the resulting GelMA retains gelatin’s unique bioactivity and biocompatibility that would enhance the cell adhesion, proliferation, and spreading thanks to its MMP-degradable amino acid sequences and RGD motif. Notably, the 3D cell-laden scaffolds of GelMA composites could be designed to emulate the native architecture of tissues and organs, thus ensuring their applications in bone tissue engineering and regenerative medicine. Moreover, GelMA composites can be prepared using other materials such as inorganic nanoparticles, carbonaceous nanomaterials, and natural/synthetic polymers. These hybrid GelMA composites display augmented mechanical properties, conductivity, and bioactivity of GelMA. Thus, GelMA composite biomaterials will endure serving as propitious bioactive candidates in bone tissue engineering and new biomedical applications. Several GelMA composites were developed for bone tissue engineering and other biomedical applications, but still, they possess several limitations. For instance, the poor mechanical properties of these composites impede their clinical applications. There is a great need to improve the mechanical properties of GelMA by combining them with other nanofillers to satisfy the demand for mechanical tolerance of biological systems. Ultimately, a significant step toward a comprehensive and practical application of GelMA composites is successful clinical translation to humans safely in a cost-effective fashion. Numerous potential GelMA composites have been advanced and tested for their ability in bone tissue engineering. However, in vivo studies of these scaffolds are still in their infancy. Eventually, it is imperative to accelerate the translation process of advanced GelMA scaffolds into clinical studies using standard protocols. Despite these disputes, GelMA scaffolds are prone to evidence of the soon evolution of bone tissue engineering and other biomedical engineering applications, with great tasks and opportunities ahead.

## Figures and Tables

**Table 1:** Recent advances of nanoparticles-integrated GelMA composites for bone tissue engineering.

Function	Type of Nanoparticle	Limitations Solved by the Nanoparticle	Target Application	Ref.
Improve physical and/or biological properties of GelMA	Sr-NPs	Most available bio-inks do not support the post-printing maturation tissue process	Nanocomposite bio-ink for 3D bioprinting	[26]
	BCP-NPs	Nanocomposites of HA or β-TCP have limitations for bone regeneration.	GelMA nanocomposite to treat significant defects in bones	[27]
	Mg-PCL	Increase physical stability and biological functionality	Nanocomposite bio-ink for 3D bioprinting	[28]
	LPN	Weak rheological properties and soft 3D structure	GelMA nanocomposite bio link	[29]
	PDA	Most photothermal agents are not suitable for mild PTT	Composite for PTT	[30]
Controlled drug release in GelMA hydrogels	Nanoliposomes	Rapidly release of drugs with GelMA	Promising bio link	[31]
	MSNs loaded with MF	MF dilutes rapidly	Injectable hydrogel for craniomaxillofacial bone regeneration.	[32]
	MSN	Some available bio-inks do not have nanosized minerals present in bones	Nanocomposite bio-ink for 3D bioprinting	[33]
Fabrication of periosteum with GelMA	CaPs	Most artificial periostea focus only on osteogenesis activity ignoring angiogenesis capability.	Artificial periosteum with osteogenesis and angiogenesis capability	[34]
	nHAMA	Most artificial periosteum focuses only on osteogenesis activity ignoring angiogenesis capability.	Artificial periosteum with osteogenesis and angiogenesis capability	[35]
Imaging GelMA scaffolds	Au-NPs	GelMA scaffolds can not be monitored once implanted in vivo. Only newly formed bones can be imaged through CT.	Contrast agents for CT imaging.	[36]
	SMNP	Photoacoustic and fluorescence imaging of cartilage scaffolds have poor resolution	Contrast agents for MRI imaging	[37]

**Table 2:** Recent advantages of nanotubes and graphene-incorporated GelMA-based nanocomposites.

Composite	Application Field	Advancement/Purpose	Reference
Nanosilver/halloysite nanotubes/gelatin methacrylate (nAg/HNTs/GelMA) hybrid hydrogel	Bone Regeneration	Prevent bacterial infection and immune response.	[44]
Carbon Nanotubes–GelMA hybrid hydrogel	Tissue Engineering	Increase the possibility of cell signaling and provide better biocompatibility.	[45]
Halloysite nanotubes (HNTs) incorporated hydrogel produced by a photopolymerization method and GelMA	Bone regeneration and bone tissue engineering.	Enhance the biocompatibility, functionality, and structure of nanotubes.	[46]
TiO_2_ nanotubes (TNT) loaded with bone morphogenetic protein 2 (BMP2) together with MA-modified gelatin (GelMA) and N-Cl modification poly (N, N′-methylene bis(acrylamide)) (PMAA-Cl)	Orthopedic Field	Inhibit non-desired osseointegration and bacterial-associated infections.	[47]
Dexamethasone (DEX) is incorporated into halloysite clay nanotubes (HNTs).	Complex tissue engineering	Boost the regenerative capacity of endogenous progenitor cells via the localized presentation of therapeutics under inflammatory conditions.	[48]
GelMA-aligned CNT hydrogels	Biomedical Field	Anisotropic electrical conductivity and enhanced mechanical properties.	[54]
GelMA-PEGDA-GO bioink	Cell differentiation	Promote chondrogenic differentiation of hMSCs	[53]
GelMA-PEGDA-GO hydrogel	Mechanical properties	Enhance compressive modulus, swelling behavior, and density of hydrogels	[55]
Ti/GO-Zn/GelMA-PBA substrates	Cell differentiation and proliferation	Improve ALP activity, ECM mineralization, and genes or protein expression	[56]
GelMA-SiGO scaffold	Bone regeneration	Enhance bone regeneration proteins formation	[57]
GQDs^(-)^-GelMA hydrogels	Cell differentiation and proliferation	Enhance bone regeneration, hMSCs proliferation, scaffold’s swelling, and degradation rate	[58]

## Data Availability

Data are contained within the article.

## Abbreviations

RGD	Arginyl-glycine-aspartic acid
BCP-NPs	Biphasic calcium phosphate nanoparticles
BMSCs	Bone Marrow Stromal Cells
BTE	Bone Tissue Engineering
Ca	Calcium
CaPs	Calcium phosphate nanoparticles
CNTs	Carbon Nanotubes
MSNs-COOH	Carboxylated MSNs
CT	Computed tomography
DEX	Dexamethasone
DEP	Dielectrophoresis
EWC	Equilibrium water content
ECM	Extracellular matrix
*E. coli*	Escherichia coli
GelMA	Gelatin methacrylate
GelMA	Gelatin methacryloyl
GO	Graphene Oxide
GQDs	Graphene Quantum Dots
GelMA/PMMA/PDA	GelMA/poly(methyl methacrylate)/polydopamine nanoparticles
Au-NPs	Gold nanoparticles
HNTs	Halloysite nanotubes
SHEDS	Human exfoliated deciduous teeth
hBM-MSC	Human bone marrow-derived mesenchymal stem cells
hPDLSCs	Human periodontal ligament stem cells
hDPSCs	Human dental pulp stem cells
hMSCs	Human mesenchymal stromal cells
HA	Hydroxyapatite
β-TCP	β-tricalcium phosphate
IPN	Interpenetrating Network
KGN	Kartogenin
LPN	Laponite
LBL technology	Layer-by-layer
Arg-UPEA	L-arginine-based unsaturated poly (ester amide)
MSNs	Mesoporous silica nanospheres
MF	Metformin
MSN	Mesoporous silica nanoparticles
Mg	Magnesium
MSCs	Mesenchymal Stem Cells
MMP	Matrix metalloproteinase
nHAMA	Methacrylated hydroxyapatite nanoparticles
SMNP	Synthetic melanin nanoparticles
Sr-GelMA	The nanocomposite of Sr nanoparticles and GelMA
nAg/HNTs/GelMA	Nanosilver/halloysite nanotubes/gelatin methacrylate
PBA	Phenylboronic acid
PMBA	Periosteum mimicking bone aid
PLC	Polycaprolactone
PEGDA	Polyethylene Glycol Diacrylate
PTT	Photothermal therapy
PDA	Polydopamine nanoparticles
PMMA	Poly(methyl methacrylate)
P	Phosphate
rGO	Reduced Graphene Oxide
SL	Stereolithography
Sr-NPs	Strontium nanoparticles
SBF	Simulated body fluid
*S. aureus*	Staphylococcus aureus
SMNP	Synthetic melanin nanoparticles
